# Fluorochloridone induces autophagy in TM4 Sertoli cells: involvement of ROS-mediated AKT-mTOR signaling pathway

**DOI:** 10.1186/s12958-021-00739-8

**Published:** 2021-04-26

**Authors:** Zhijing Ni, Weiqi Sun, Rui Li, Mingjun Yang, Fen Zhang, Xiuli Chang, Weihua Li, Zhijun Zhou

**Affiliations:** 1 School of Public Health/MOE Key Laboratory for Public Health Safety/NHC Key Laboratory of Health Technology Assessment, Fudan University, Shanghai, 200032 China; 2 Shanghai Institute for Food and Drug Control, Shanghai, 201203 China; 3 NHC Key Laboratory of Reproduction Regulation (Shanghai Institute of Planned Parenthood Research), Shanghai, 200032 China

**Keywords:** Fluorochloridone, Reproductive toxicity, Autophagy, TM4 Sertoli cells, ROS, AKT /mTOR signaling pathway

## Abstract

**Background:**

Fluorochloridone (FLC), a selective pyrrolidone herbicide, has been recognized as a potential endocrine disruptor and reported to induce male reproductive toxicity, but the underlying mechanism is unclear. The aim of this study was to investigate the mechanism of FLC-induced reproductive toxicity on male mice with particular emphasis on the role of autophagy in mice’ TM4 Sertoli cells.

**Methods:**

Adult C57BL/6 mice were divided into one control group (0.5% sodium carboxymethyl cellulose), and four FLC-treated groups (3,15,75,375 mg/kg). The animals (ten mice per group) received gavage for 28 days. After treatment, histological analysis, sperm parameters, the microstructure of autophagy and the expression of autophagy-associated proteins in testis were evaluated. Furthermore, to explore the autophagy mechanism, TM4 Sertoli cells were treated with FLC (0,40,80,160 μM) in vitro for 24 h. Cell activity and cytoskeletal changes were measured by MTT assay and F-actin immunofluorescence staining. The formation of autophagosome, accumulation of reactive oxygen species (ROS), expression of autophagy marker proteins (LC3, Beclin-1 and P62) and AKT-related pathway proteins (AKT, mTOR) were observed. The ROS scavenger N-acetylcysteine (NAC) and AKT agonist (SC79) were used to treat TM4 cells to observe the changes of AKT-mTOR pathway and autophagy.

**Results:**

In vivo, it showed that FLC exposure caused testicular injuries, abnormality in epididymal sperm. Moreover, FLC increased the formation of autophagosomes, the accumulation of LC3II/LC3I, Beclin-1 and P62 protein, which is related to the degradation of autophagy. In vitro, FLC triggered TM4 cell autophagy by increasing the formation of autophagosomes and upregulating of LC3II/LC3I, Beclin-1 and P62 levels. In addition, FLC induced ROS production and inhibited the activities of AKT and mTOR kinases. The Inhibition of AKT/mTOR signaling pathways and the activation of autophagy induced by FLC could be efficiently reversed by pretreatment of NAC. Additionally, decreased autophagy and increased cell viability were observed in TM4 cells treated with SC79 and FLC, compared with FLC alone, indicating that FLC-induced autophagy may be pro-death.

**Conclusion:**

Taken together, our study provided the evidence that FLC promoted autophagy in TM4 Sertoli cells and that this process may involve ROS-mediated AKT/mTOR signaling pathways.

## Introduction

Fluorochloridone (FLC), a selective pyrrolidone herbicide, is used for the control of various broad-leaved weeds. FLC had medium persistence in soil and groundwater, indicating that its environmental fate was highly correlated with mammals and human health. Our previous experiments revealed that the target cell was Sertoli cell with pathological characteristic of vacuolation, which may induce adverse effects in the reproductive functions and hormonal systems of male rats [1]. Notably, we also confirmed that FLC induced apoptosis of Sertoli cells and the loss in BTB integrity, which cause testis abnormally and affect fertility [2]. FLC induced testis injuries in rats, however, there still exist data gaps to mice. We aimed to study the mechanism of FLC-induced reproductive toxicity on male mice and investigate on the role of autophagy in mice’ TM4 Sertoli cells.

Sertoli cell lines comprise a good model for evaluating the mechanism of toxin-induced testis damages. Sertoli cells orchestrate the processes of spermatogenesis by providing nutrition for developing germ cells, and are absolutely crucial for germ cell development and viability [3]. Impairment of Sertoli cells function by many environmental chemical toxicants may compromise spermatogenesis and hence male fertility [4].

Autophagy, a process of programmed cell death, is a highly conserved metabolic process that provides nutrients and energy cell repair and reconstruction by degrading abnormal macromolecular proteins and organelles [5]. During autophagy, some of the cytoplasmic proteins and organelles are sequestered into double membrane vesicular formations that fuse with the lysosomes to degrade their contents [6]. During autophagy induction, the conversion of LC3-I to LC3-II and the formation of autophagosomes are hallmark features of autophagy [7]. Autophagy is usually a cell survival mechanism; however, its extension could result in type II programed cell death or autophagic cell death [8]. Therefore, it is necessary to clarify the exact role of autophagy.

Oxidative stress, including ROS production, is considered to be the initiating factor of autophagy. Under normal physiological conditions, the production and clearance of ROS are maintained at a stable level and regulated by oxidation and antioxidant systems in the organism, while excessive amounts of ROS induce cell autophagy [9]. In addition, ROS was shown to play a regulatory role in autophagy modulating the AKT-mTOR signaling pathway [10]. The protein kinase B (AKT)/mammalian target of rapamycin (mTOR) pathway, a classic autophagy pathway [11], acts as a crucial part in cell growth, proliferation and autophagy.

Our previous studies have shown that FLC exposure induces apoptosis of Sertoli cells, but whether FLC can induce autophagy, and the underlying mechanism of autophagy in Sertoli cells, remain unclear. In the present study, we investigated the effects of FLC on reproduction and explored the active states of ROS-mediated AKT/mTOR signaling pathways and relationship with autophagy. The results will provide novel clues to reveal the reproductive toxicity mechanism of FLC.

## Methods

### Experiment animals and treatment

Adult Six-week-old male C57BL/6 mice was obtained from the Shanghai Lingchang Biotechnology Co., LTD (Shanghai, China). The use of animals was in accordance with the Guidelines for the Care and Use of Laboratory Animals issued by the Ministry of Health of the People’s Republic of China. The animals were housed under standard laboratory conditions at constant temperature (21–24 °C) and humidity (40–70%) on 12-h light/dark cycles. Standardized granular diet and water were available.

After 1 week of acclimatization, mice were randomly divided into five groups of ten mice each: four FLC treatment groups and a vehicle control group (0.5% CMC-Na). Based on our preliminary experiment and the data showed by European Food Safety Authority (EFSA) that rats were more sensitive than mice [12], we selected finally the dose of 3, 15, 75 and 375 mg/kg in this study. The mice administered gavage once a day for 28 consecutive days.

### Cell culture and treatment

Mouse TM4 Sertoli cells were obtained by Chinese Academy of Sciences. Cells were cultured at 37 °C in 5% CO2 in Dulbecco’s Modified Eagle Medium/F12 supplemented with 10% fetal bovine serum and antibiotics (0.5% penicillin G -streptomycin). FLC was dissolved in DMSO and diluted with DMEM medium to final concentrations (40, 80 and 160 μM). For intervention experiments, cells were pretreated with NAC (5 mM for 2 h), SC79 (20 μM for 2 h) followed by treatment with 160 μM FLC.

### Epididymis sperm analysis

After the mice of each group were decapitated and sacrificed, the epididymal tail was immediately taken out and placed in warm phosphate-buffered saline at 37 °C for washing, and then placed in the preheated sperm nutrient solution (37 °C, pH = 7.4) by mincing the cauda epididymis. Sperm count and motility were determined by computer assisted semen analysis (CASA).

### Testicular histopathology

After fixation with 4% paraformaldehyde for 24 h, the fixed testis was embedded in paraffin blocks cut into 10 μm sections. The sections were stained with HE according to a standard protocol. The stained sections were mounted and examined under a light microscopy.

### Measurement of cell viability by MTT assay

Cells were seeded into 96-well plates and adhered overnight. Cells were treated with allicin (40, 80, 120, 160 and 200 μM) for 24 h. Afterwards, cells were added with 10 μl MTT solution and incubated for 4 h at 37 °C. After the medium was removed, 150 μl of DMSO was added to each well to dissolve the formed formazan crystals. Cell viability was measured at 490 nm using a spectrometer.

### F-actin staining

Cells were fixed in 4% paraformaldehyde and incubated with FITC-conjugated phalloidin at 1:50 dilution for 1 h, to visualize F-actin. Mounted with DAPI to visualize cell nuclei. The fluorescence images were recorded using Inverted Fluorescence Microscope.

### Determination of ROS

Intracellular ROS levels were measured by using a reactive oxygen species assay kit according to the manufacturer’s protocol. Cells were incubated with the fluorescent probe 2, 7-dichlorodihydrofluorescindiacetate (DCFH-DA) (1: 1000) at 37 °C in the dark for 30 min, and then fluorescence microscopy was observed and photographs were taken.

### Detection of autophagosome formation

For ultrastructural studies, testis and TM4 Sertoli cells were pelleted and fixed in 2.5% phosphoric acid buffer solution of glutaraldehyde, subsequent procedures were performed using standard methods. The ultrastructure of testis and cells were observed with a transmission electron microscope.

### Measurement of autophagy vesicles by MDC staining

For quantitative assessment of the late-stage autophagosomes, autophagic vacuoles were stained with MDC for autophagy analysis as previously described [13]. Briefly, after treatment, cells were incubated with MDC for 45 min and collected by centrifugation, and finally MDC-positive cells were quantified by flow cytometry.

### Western blot analysis

Briefly, the proteins of testis and cells were extracted using the RIPA lysis buffer and mixed with loading buffer were collected and boiled for 5 min. The protein was extracted and electrophoresed on SDS-polyacrylamide gel, and then transferred onto a PVDF membrane. The PVDF membrane was incubated with primary antibodies against, p-AKT, AKT, p-mTOR, mTOR, LC3, Beclin1, P62 and GADPH (Cell Signaling Technology, Inc.) at 1: 1000 dilutions overnight at 4 °C. The membranes were incubated with 5% non-fat milk for 2 h at room temperature and then incubated with primary antibodies overnight, followed by peroxidase-conjugated secondary antibody for 1 h at room temperature. The protein signals were detected by enhanced chemiluminescence (ECL) detection system and analyzed by using ImageJ.

### Statistical analysis

All experiments were repeated independently at least in triplicate. The normality test showed that our measurement data obeyed a normal distribution. The values were expressed as the mean ± SD (standard deviation). The data from multiple groups were performed using one-way ANOVA, whereas pairwise comparisons between two groups were compared using t-tests. Ns represents no significance. *P < 0.05, **P < 0.01 and ***P < 0.001 were considered statistically significant.

## Results

### Effects of FLC exposure on mice testis

**Fig. 1:**
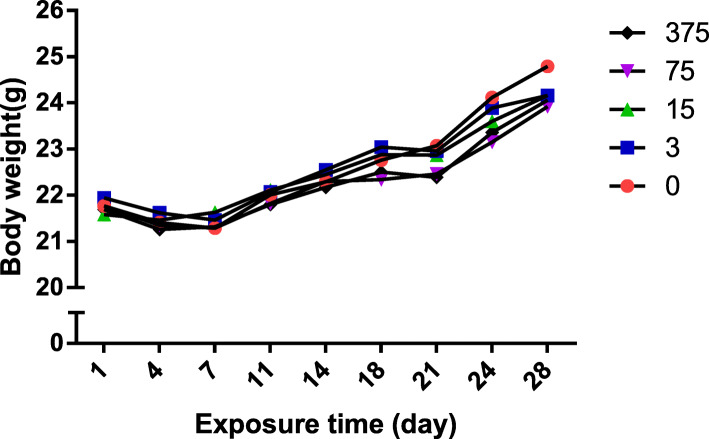
Male body weight changes during Administration Day (AD), *n* = 10 in each group

**Fig. 2:**
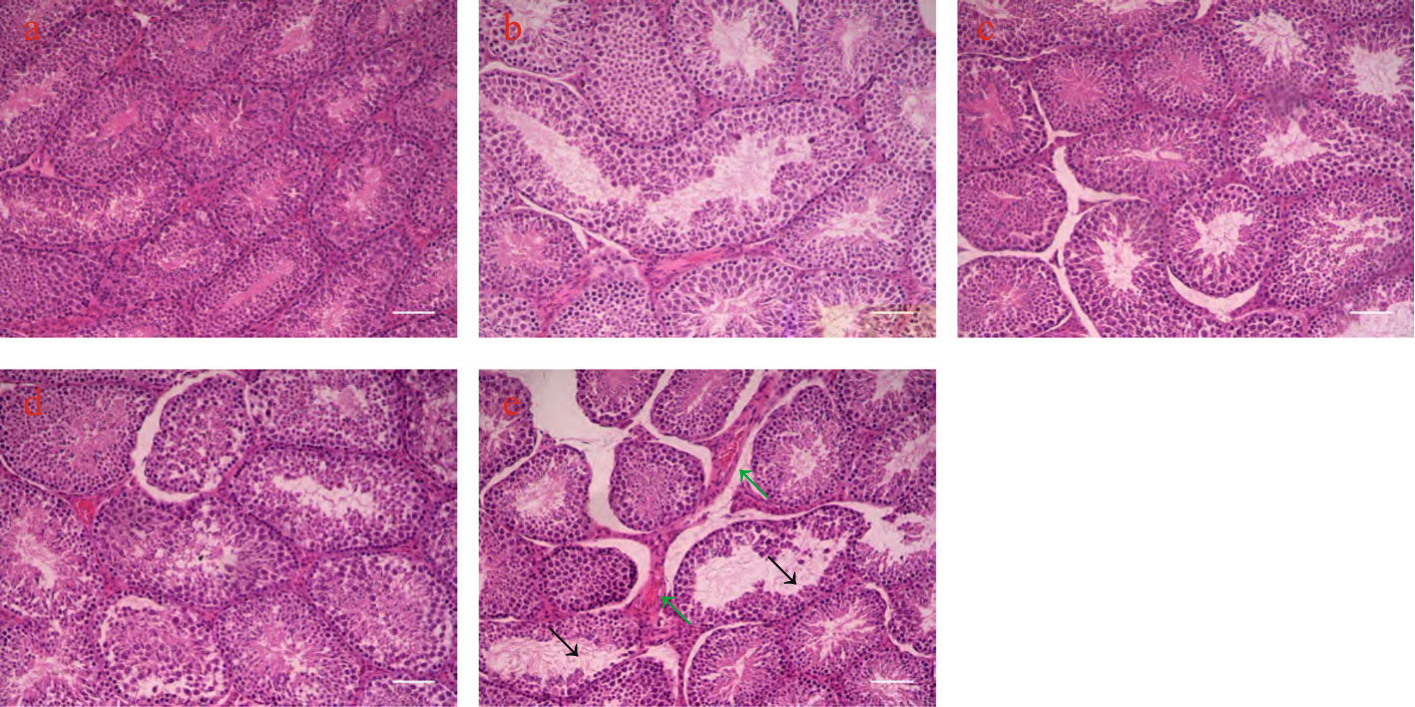
Pathological injuries after 28d FLC exposure in testis (200×). The reduction of spermatogenic cells caused by FLC with a black arrow, and the emergence of fibroblasts with a green arrow. **a** control; **b** 3 mg/kg FLC group; **c** 15 mg/kg FLC group; **d** 75 mg/kg FLC group; **e** 375 mg/kg FLC group

There was a significant decrease in organ coefficient from 375 mg/kg/day FLC exposure (Table 1), though no significant decrease in body weights was observed (Fig. 1). The findings revealed the sperm motility decreased after 75 mg/kg/day FLC exposure. Notably, the sperm motility and count decreased after 375 mg/kg/day FLC exposure (Table 1). Testicular pathological examination showed that FLC exposure injured the testicular structure by reducing spermatogenic cells and the emergence of fibroblasts in the interstitium (Fig. 2).

**Table 1:** Reproductive testicular coefficient and sperm examination of male mice (mean ± SD)

Parameters	Control *n* = 10	3 mg/kg *n* = 10	15 mg/kg *n* = 10	75 mg/kg *n* = 10	375 mg/kg *n* = 10
Testicular coefficient (%)	0.73 ± 0.06	0.79 ± 0.04	0.76 ± 0.07	0.76 ± 0.06	0.65 ± 0.04**
Sperm count (M/ml)	30.20 ± 6.32	30.60 ± 5.12	26.58 ± 6.56	24.90 ± 7.83	23.12 ± 5.89*
Sperm motility rate (%)	69.33 ± 8.34	64.25 ± 15.92	62.75 ± 16.88	55.57 ± 14.51*	55.57 ± 12.49*

Testicular coefficient: (testes weight/body weight) × 100

*P < 0.05; **P < 0.01 significantly different from the corresponding group

**Fig. 3:**
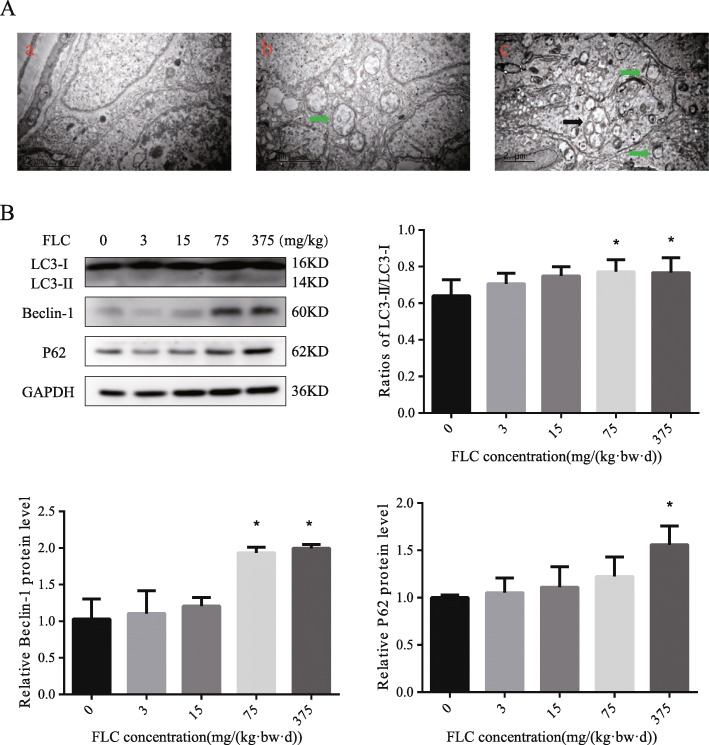
Effect of FLC on testicular autophagy. **A**, Testicle tissue sections were analyzed by TEM. Green arrowheads represent autophagy precursors; black arrows point at mature autophagic vesicles with mitochondria. (a) control; (b) 75 mg/kg FLC group; (c) 375 mg/kg FLC group. **B**, The protein levels of LC3, Beclin-1, and P62 were determined by western blotting. *P < 0.05; **P < 0.01 significantly different from the corresponding group

TEM analysis found that autophagosomes with membranous structures was presented clearly in the 375 mg/kg/day FLC exposure group, which suggested that FLC could promote Sertoli cell autophagy in testis (Fig. 3A). After 28 days of FLC exposure, compared with in the control, the expression levels of LC3II/I and Beclin-1 significantly increased in the 75 mg/kg/day FLC exposure groups. The expression level of LC3II/I、Beclin-1 and P62 in the 375 mg/kg/day FLC exposure groups was also increased (Fig. 3B), which indicated that FLC might induce occurrence of autophagy in testis.

### FLC-induced changes in TM4 Sertoli cell viability, morphology and autophagy

**Fig. 4:**
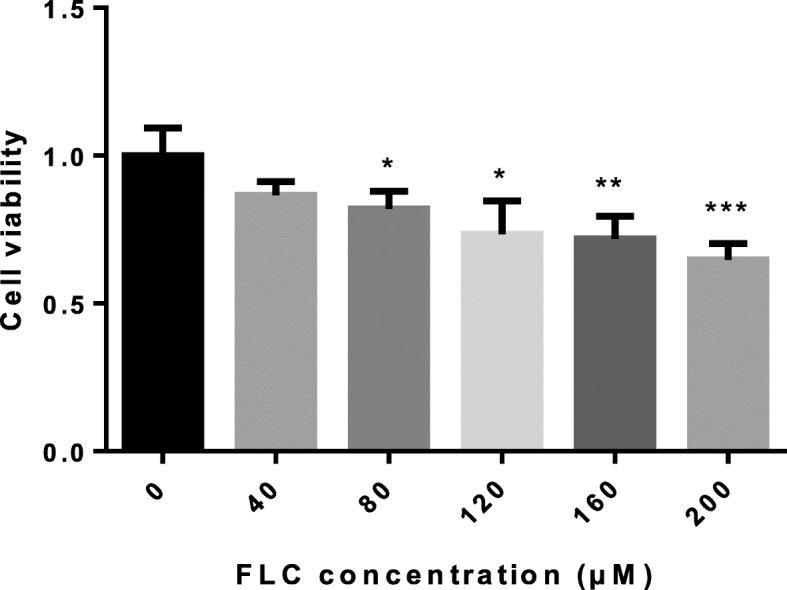
MTT assay was performed to evaluate TM4 Sertoli cell viability treated with different concentrations (0, 40, 80, 120,160,200 μM) of FLC for 24 h. *P < 0.05; **P < 0.01, *** *P* < 0.001 significantly different from the corresponding group

MTT assay was applied to test the inhibitory effect of FLC on changing cell viability of cells in vitro. The cells were, respectively, treated with different concentrations (0, 40, 80, 120, 160, 200 μM) of FLC for 24 h.The results of an MTT assay also revealed that FLC exhibited an anti-proliferative effect on cells and significantly suppressed the growth of cells in a dose-dependent manner (Fig. 4). In order to carry out follow-up studies, the control group and FLC exposure group (FLC concentrations of 40, 80 and 160) were set respectively in this study.

**Fig. 5:**
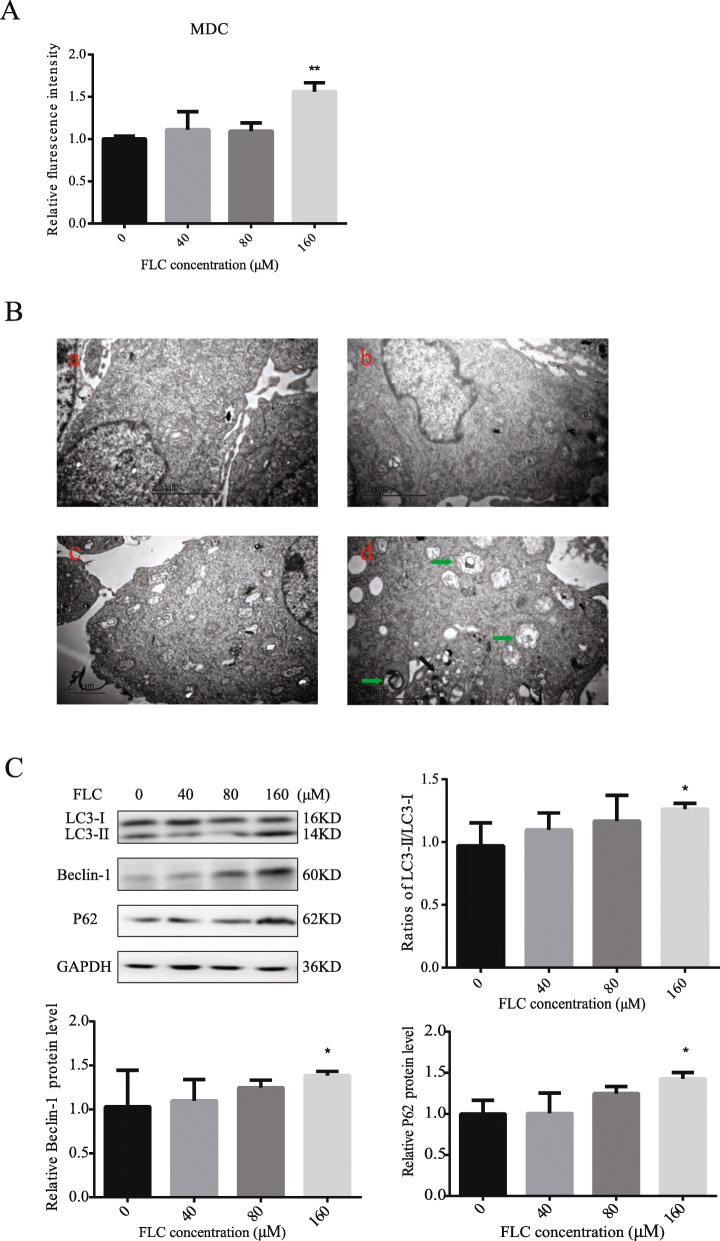
Effects of FLC treated in TM4 Sertoli cells autophagy activation. **A**, Flow cytometric analysis of MDC-labeled autophagic vesicles. **B**, Representative transmission electron micrographs depicting ultrastructure of TM4 Sertoli cells following 24 h treatment with FLC. Green arrowheads indicate the typical autophagosomes containing intracellular components and organelles; black arrowheads indicate the lysosomal. (a) control; (b) 40 μM FLC group; (c) 80 μM FLC group; (d) 160 μM FLC group. **C**, Western blot of autophagy-activating proteins (LC3, Beclin-1, P62). *P < 0.05 significantly different from the corresponding group

**Fig. 6:**
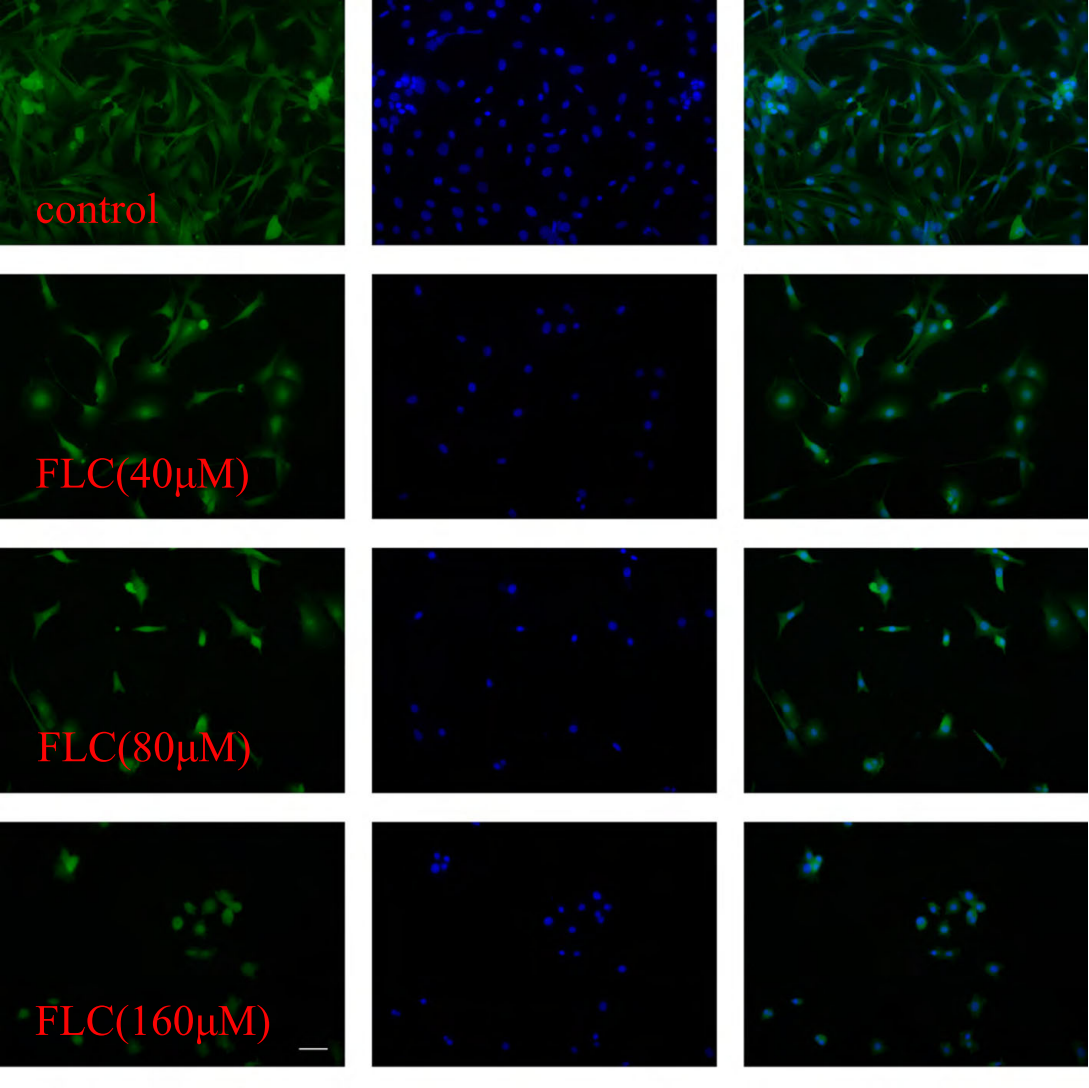
Effects of FLC treatment on F-actin organization

MDC immunostaining was used to confirm autophagy induction by FLC. Compared with the control group, FLC treatment at 160 μM increased the fluorescence intensity of cell indicating extensive MDC-positive autophagy (Fig. 5A). As illustrated in Fig. 5B, we noted that FLC stimulated the formation of lysosomes as well as autophagosomes. We observed more membrane whorls and vacuoles containing degraded organelles, and swollen mitochondria devoid of cristae. we next investigated the potential effects on the expression of autophagy-activating proteins. Compared to the control group, 160 μM FLC treatment significantly increased the expression of LC3-II/I, Beclin-1 and P62(Fig. 5C). In order to visualize the changes in the cytoskeleton, F-actin was stained with FITC-conjugated phalloidin. Compared to the control group, 160 μM FLC treatment not only reduced the length of microfilament but also inhibited the microfilament branching. Normal Sertoli cells are Conical and narrow, FLC treatment at 160 μM for 24 h caused abnormal morphological changes, including cell shrinkage and appearance of floating cells (Fig. 6).

### FLC triggered Reactive oxygen species(ROS) accumulation and inhibited AKT signaling pathway

**Fig. 7:**
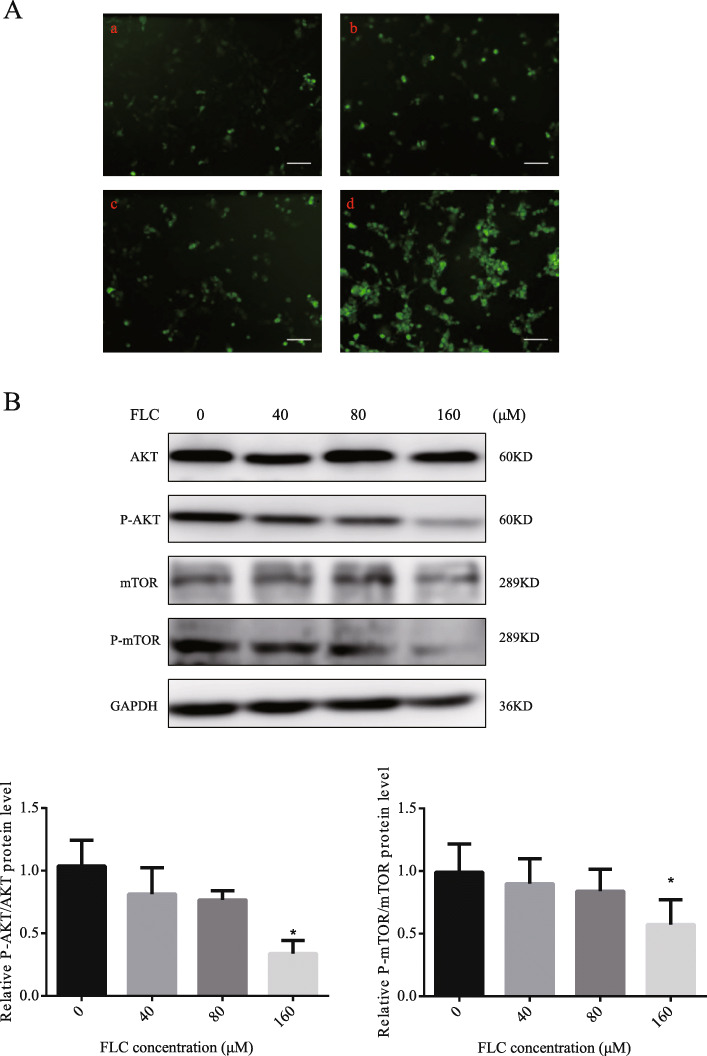
The effect of FLC on ROS accumulation, AKT/mTOR signaling at 24 h. **A**, Effect of different concentrations of FLC on the accumulation of ROS. (a) control; (b) 40 μM FLC group; (c) 80 μM FLC group; (d) 160 μM FLC group. **B**, Western blot of AKT/mTOR signaling pathway proteins (AKT, p-AKT, mTOR, p-mTOR). *P < 0.05 significantly different from the corresponding group

2, 7-dichlorodihydrofluorescein (DCFH) staining was conducted to detect the level of ROS in TM4 Sertoli cells. FLC dose-enhanced diffuse cytosolic labeling (Fig. 7A). The result indicating that FLC triggered ROS accumulation. To further investigate the mechanism of FLC-induced TM4 Sertoli cells autophagic cell death, we examined the expression level of AKT, p-AKT, mTOR, and p-mTOR, As showed in Fig. 7B, the expression levels of p-AKT/AKT, p-mTOR/ mTOR were decreased in a concentration-dependent manner.

### NAC effectively reversed the inhibition of AKT signaling pathway and autophagy activation

**Fig. 8:**
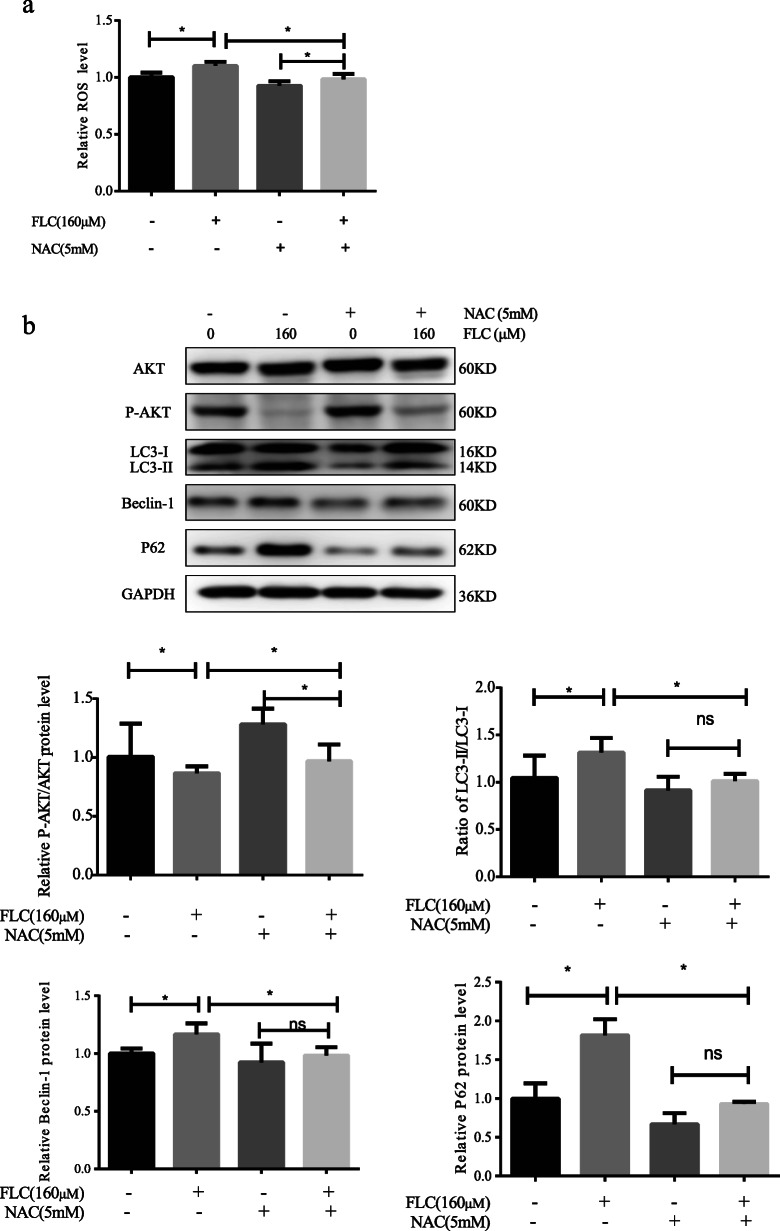
The role of NAC in FLC-induced ROS levels, AKT activation and autophagy levels. Sertoli cells were pretreated with 5 mM NAC for 2 h and then exposed to FLC. **a** The effect of FLC-induced ROS level was observed. **b** Western blot detected AKT signaling pathway proteins (AKT, p-AKT) and autophagy-activating proteins (LC3, Beclin-1, P62). Ns represents no significance, *P < 0.05 significantly different from the corresponding group

To determine the role of ROS in FLC-induced TM4 Sertoli cell autophagy,5 mM NAC was used to pretreat the cell samples for 2 h prior to FLC treatment. NAC reduced the FLC-induced ROS level elevation and the expression of LC3II/LC3I, Beclin-1 and P62(Fig. 8a, b). Importantly, NAC increased the expression of p-AKT/AKT, which suggested that ROS was likely to be the upstream signal molecule of AKT signaling (Fig. 8b). The results showed that NAC pretreatment reversed TM4 Sertoli cells autophagy induced by FLC.

### SC79 inhibits FLC-induced autophagy and increased cell viability

**Fig. 9:**
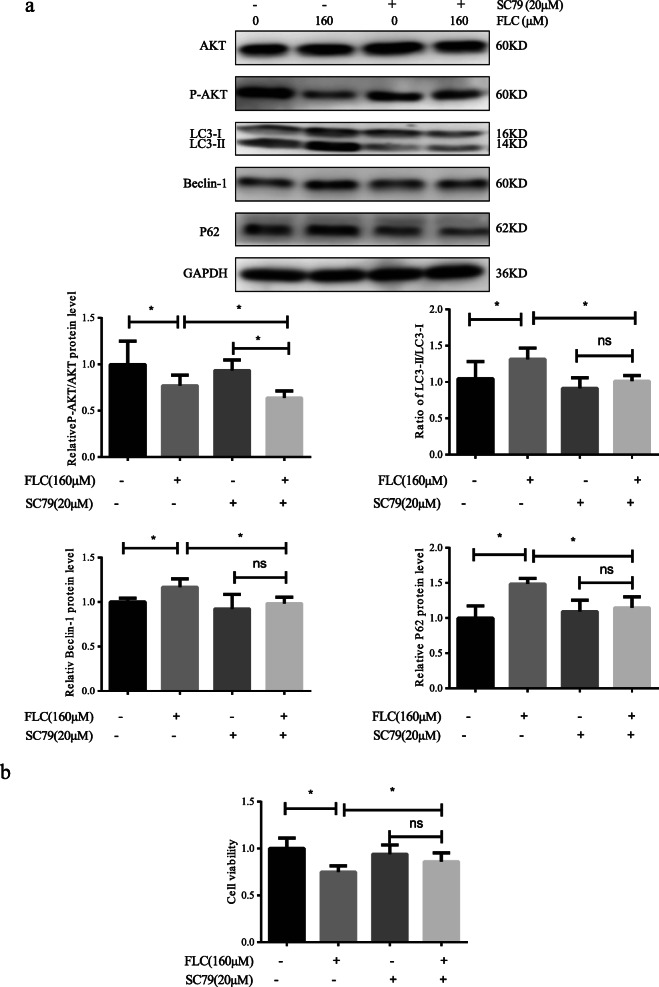
The role of SC79 in FLC-induced AKT activation, autophagy levels and cell viability. TM4 Sertoli cells were pretreated with 20 μM SC79 for 2 h and then exposed to FLC. **a** Western blot detected AKT signaling pathway proteins (AKT, p-AKT) and autophagy-activating proteins (LC3, Beclin-1, P62). **b** Cell viability as determined by using MTT assay. Ns represents no significance, *P < 0.05 significantly different from the corresponding group

To determine whether FLC-induced autophagy is related to AKT/mTOR signaling pathway, we used 20 μM SC79, the AKT agonist, to pretreat the cell samples for 2 h prior to FLC treatment. The overexpression of AKT increased expression of p-AKT/AKT and reduced expression of LC3II/LC3I, Beclin-1 and P62(Fig. 9a). The date indicated that the overexpression of AKT reversed FLC-induced autophagy. Moreover, the MTT assay result showed that the inhibited cell growth effects were decreased when treatment with FLC was combined with SC79 (Fig. 9b). Our study showed that combined treatment with an established AKT agonist increased FLC-induced cell viability.

## Discussion

In vivo, adverse changes in count and motility of epididymal sperm and damage to the testicular structure by reducing spermatogenic cells were found after FLC administration, suggesting that FLC disturbs spermiogenesis. Together, these observations indicated that FLC could trigger testicular functional deterioration, stimulating us to further investigate its mechanism.

Varicoceles [8, 14] and high production of ROS [15–17] are common cause of male infertility. Our previous studies have shown that FLC can reduce sperm quality by inducing oxidative stress [18]. Accumulating evidence also indicated that autophagy plays a regulatory role in the damage of spermatogenesis [19]. This article mainly investigated whether autophagy was involved in the process of spermatogenesis in vivo. In the current study, we observed mature autophagosomes in Sertoli cells through electron microscopy after FLC exposure. Based on this, we found that FLC triggered autophagy and elevated the expression of Beclin-1 and LC3 in testis. Interestingly, the expression of P62 also increased, as an indication of autophagic flux, which indicated that autophagy degradation is impaired. Furthermore, we speculated that autophagy may be involved in the process of spermatogenesis, however, the underlying mechanism was not clear.

To investigate the mechanism of autophagy, we established an in vitro model of the TM4 Sertoli cells. The results of MTT assay revealed that the cell viability gradually decreased with the increase of the FLC dose, indicating FLC could suppress cell proliferation and had potential cytotoxicity. In addition, we found that autophagy was induced by FLC as evidenced by the accumulation of autophagic vesicles and upregulation of the protein expression of LC-3, Beclin-1 and P62. Studies have revealed that F-actin polymerization assists autophagosome–lysosome fusion [20]. Our experiments show that the FLC is destroyed the actin filaments. The combination of the increase of P62 and the destruction of F-actin indicated that the fusion of autophagosome and lysosome is blocked.

One of the striking features of the autophagy in Sertoli cells is the generation of ROS. ROS forms as natural byproduct of the normal metabolism of oxygen and plays a critical role in inducing both cell apoptosis and autophagy [21]. Our present data showed that FLC induced a significant increase in ROS generation. NAC, the ROS scavenger, remarkably decreased the expression level of LC-3, Beclin1 and P62. All these results indicate that FLC triggered autophagy directly or indirectly by inducing the accumulation of intracellular ROS, but its exact mechanism needs to be studied in depth.

Recent studies have shown that multiple signaling molecules, such as MAPKs, AMPK, and class III PI3K, have been related to apoptosis and autophagy [22, 23]. AKT /mTOR signaling pathway is the most classic pathway to trigger autophagy, so we focused on whether FLC-induced intracellular ROS accumulation can induce autophagy through the AKT /mTOR signaling pathway. Our current results showed that FLC triggered the inhibition of AKT and mTOR. Moreover, NAC could effectively antagonize the low expression of ^Ser473^p-AKT/AKT induced by FLC. In addition, SC79, AKT agonist, significantly decreased the expression of LC-3, Beclin-1 and P62, which could restore the autophagy induced by FLC in TM4 Sertoli cell. Duan, P [24] found Nonyl Phenol promotes autophagy in TM4 Sertoli cells and that this process may involve ROS-dependent AKT /mTOR pathways, which is consistent with our study. Combined treatment with an established AKT agonist increased FLC-induced cell viability, which indicated that FLC-induced autophagy may be pro-death. Zhang, Y found that the autophagy activator Rapa alleviated intracellular ROS and Bisphenol A -driven cell viability [25], which similar to our study. Taken together, all the above results suggested that FLC triggered autophagy which could be mediated by the activation of ROS and the inhibition of AKT/mTOR signaling pathways.

## Conclusion

In conclusion, we demonstrated that FLC induced Sertoli cells autophagy in vivo and vitro. FLC induced autophagy through the activation of ROS and the inhibition of AKT/mTOR signaling pathways. Moreover, AKT agonist restore the autophagy and increased FLC-induced cell viability, indicating that FLC-induced autophagy is a pro-death process in cells. Our findings not only put further insights into the potential mechanisms of FLC, but also provide a strategy for therapy.

## Data Availability

All data supported the conclusions during this study are included within the article.

## Abbreviations

FLC: Fluorochloridone
CMC-Na: Sodium carboxymethyl cellulose CMC-Na
TEM: Transmission electron microscopy
CASA: Computer assisted semen analysis
DMSO: Dimethyl sulfoxide
NAC: N-acetyl-l-cysteine
MTT: 3-(4,5-dimethylthiazol-2-yl)-2,5-diphenyltetrazolium bromide
MDC: Monodansylcadaverine
ROS: Reactive oxygen species
RIPA: Radio Immunoprecipitation Assay
